# Accurate and scalable representation of electric vehicles in energy system models: A virtual storage-based aggregation approach

**DOI:** 10.1016/j.isci.2023.107816

**Published:** 2023-09-01

**Authors:** Jarusch Muessel, Oliver Ruhnau, Reinhard Madlener

**Affiliations:** 1 Potsdam Institute for Climate Impact Research, Potsdam, Germany; 2 Global Energy Systems Analysis, Technical University Berlin, Germany; 3 Department of Economics and Institute of Energy Economics, University of Cologne, Germany; 4 Hertie School, Berlin, Germany; 5 Institute for Future Energy Consumer Needs and Behavior (FCN), School of Business and Economics/E.ON Energy Research Center, RWTH Aachen University, Germany; 6 Department of Industrial Economics and Technology Management, Norwegian University of Science and Technology (NTNU), Trondheim, Norway

**Keywords:** Energy resources, Energy systems, Energy Modelling

## Abstract

The growing number of electric vehicles (EVs) will challenge the power system, but EVs may also support system balancing via smart charging. Modeling EVs’ system-level impact while respecting computational constraints requires the aggregation of individual profiles. We show that studies typically rely on too few profiles to accurately model EVs’ system-level impact and that a naïve aggregation of individual profiles leads to an overestimation of the fleet’s flexibility potential. To overcome this problem, we introduce a scalable and accurate aggregation approach based on the idea of modeling deviations from an uncontrolled charging strategy as virtual energy storage. We apply this to a German case study and estimate an average flexibility potential of 6.2 kWh/EV, only 10% of the result of a naïve aggregation. We conclude that our approach allows for a more realistic representation of EVs in energy system models and suggest applying it to other flexible assets.

## Highlights

• We introduce a scalable and accurate aggregation approach for electric vehicles (EVs)
• Our approach allows for a more realistic representation of EVs' flexibility in energy system models
• The core idea is to model deviations from an inflexible reference as a virtual energy storage
• We estimate an average flexibility potential of 6.2 kWh/EV for our German case study

## Graphical abstract

**Figure:**
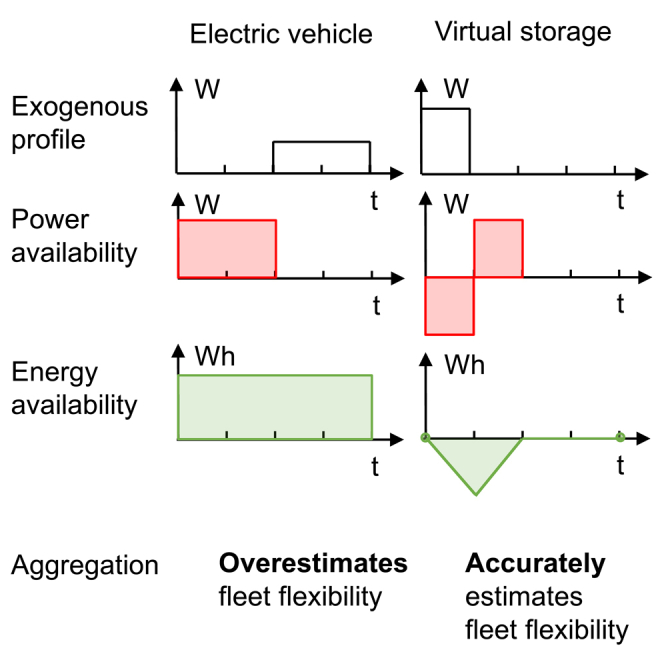

## Introduction

The electrification of passenger cars is crucial for fighting against climate change. The transport sector accounted for 37% of global CO_2_ emissions of end-use sectors in 2021, 40% of which can be attributed to passenger cars.^1^ Direct electrification is the most energy-efficient option for most mobility applications and, with renewable energy sources driving the decarbonization of the power sector, a key solution for transport decarbonization.^2^ As a result, electric vehicles (EVs) play a major role in decarbonization scenarios. In Germany, for example, the number of EVs is projected to increase to 15 million by 2030, from about 1.5 million today (approx. 0.8 million battery electric vehicles and 0.7 million plug-in hybrids).^3^ Note that future scenarios include mainly battery EVs, and we therefore focus on those, rather than on plug-in hybrids throughout this article.

The charging of EVs may introduce substantial volatility to the power system. As the primary use of EVs is mobility, charging behavior mainly follows mobility requirements. In the extreme case of uncontrolled charging, this would imply charging with the maximally accessible power rating as soon as energy is available until the battery is full. For the example of Germany, the expected increase in EVs in 2030 would correspond to an increase in electricity consumption from 5 today to 53 TWh in 2030, i.e., about 10% of today’s aggregate electricity demand.^4^ The effects on the peak electricity load are even larger. Earlier studies found that the uncontrolled charging of 8 million EVs could cause significant peak load increases,^5^ e.g., of 34 GW,^5^^,^^6^^,^^7^ compared with the current overall German peak load of 70–80 GW.^8^ The impact of 15 million EVs in 2030 on the system balance can be expected to be even more severe. In addition, the volatility of increasing renewable electricity generation fosters the potential temporal mismatches between supply and demand even more, calling for flexibility solutions.^9^

At the same time, EVs could also have a balancing effect on the power system during their idle times of above 90%.^10^^,^^11^ As EVs can shift loads via smart charging and even feed electricity back into the grid via bidirectional charging (also referred to as vehicle-to-grid), they can provide short- to mid-term flexibility to the power system.^12^^,^^13^ So far, no general definition of flexibility has emerged, and its meaning depends on the context.^14^^,^^15^^,^^16^ In this article, flexibility is understood in the context of electrical systems as the potential to shift energy consumption as well as storing and later discharging energy when required. According to the literature, exploiting EVs’ flexibility potentials could entail a peak load reduction of more than 17%.^17^^,^^18^ In particular, EVs could contribute to the feasibility of highly renewable electricity scenarios by offering their flexibility to the system and substituting for increasing energy storage requirements.^19^^,^^20^^,^^21^^,^^22^

The accurate representation of the volatility and flexibility of EVs in energy system analyses is both critical and challenging. Due to computational constraints, a fleet of millions of EVs cannot be modeled individually but must be approximated by a representative subsample. Previous studies did so based on 50 to 200 profiles,^23^^,^^24^ but it remains unclear how many profiles are needed for an adequate representation on a system level. In addition, even if the number of profiles is reduced to a representative subsample, the inclusion in complex energy system models may require their aggregation into one profile for the entire EV fleet.^25^^,^^26^^,^^27^^,^^28^ Previous studies discuss that existing aggregation approaches used for energy system modeling overestimate the aggregated flexibility potential.^29^ Although other studies have proposed more accurate aggregation algorithms for the optimization of EV charging, these are based on exogenous steering signals, such as prices or volume schedules.^28^^,^^30^^,^^31^ Consequently, they cannot be integrated into energy system models where the dispatch of EVs is jointly optimized with the dispatch of other technologies and prices are determined endogenously.

This article makes three contributions to overcome the mentioned modeling challenges. First, we explain and quantify the discussed representativity and aggregation problem. Second, we derive the minimum number of individual EV profiles required to fulfill a given accuracy target. Third, and most importantly, we propose a solution to the aggregation problem that allows to generate an accurate fleet-level flexibility profile that can be implemented into large-scale energy system models with endogenous prices and dispatch.

Our approach bases on the concept of a virtual energy storage. In energy system modeling, the concept of virtual power plants as a generation technology is widely used,^32^^,^^33^ but the virtual energy storage as we define and apply it in this article remains a novel concept. The main idea behind the virtual energy storage approach is that the EV’s flexibility potential is modeled as the deviation from an uncontrolled charging strategy while respecting its primary purpose: mobility. This method allows to accurately aggregate many single profiles into one fleet profile. We apply this approach to a case study with 15 million EVs, which is the projection for Germany in 2030, represented by a simulated dataset of 12,000 individual consumption and power availability profiles.

The remainder of this article is structured as follows. First, we explain how we determine a representative sample size for modeling an EV fleet. Second, we elaborate on why current aggregation approaches overestimate flexibility potentials. Subsequently, we introduce our main methodical contribution, the virtual energy storage approach, and apply it to our German case study. In two final sections, we discuss our results and draw conclusions. Detailed explanations are provided in the attached STAR methods section and the supplementary information.

## Results

### Why many profiles are needed for a representative EV fleet

We first aim to determine how many profiles are needed to representatively model a large EV fleet. We do so based on synthetic load profiles because empirical profiles are scarce and associated with significant limitations and uncertainties.^34^ The load profiles of individual EVs were synthesized with the stochastic tool *emobpy*,^23^ which is based on empirical mobility statistics up until 2019. In this article’s case study, we make assumptions on EV diffusion based on projections for 2030, as described in Note S1 and further discussed by Muessel et al.^34^ One critical assumption, which is discussed further below, is a plug-in rate of 100%, which means that EVs are always connected to charging stations when they are available. Furthermore, we start by assuming an uncontrolled charging strategy, which is then varied later on in the article.

We determine the representativity based on After Diversity Maximum Demand (ADMD), which is an established metric that calculates the per-consumer peak load of a given consumer group (see Note S2 and work by Sun et al.^35^). In general, representativity implies that the sampled fleet converges in its behavior to the underlying probability distribution.^11^ Using the ADMD as an indicator, we focus on the fleet’s peak charging demand, which is critical for system-level questions such as capacity planning. Modeling a large fleet based on too few individual profiles may lead to an overestimation of the peak load.^36^ This is because diverse consumption patterns imply that with increasing numbers of customers, the maximum time-coincident demand per consumer falls.^37^ Here, we calculate the ADMD for fleets of 10,–12,000 EVs over a period of three months.

**Figure 1:**
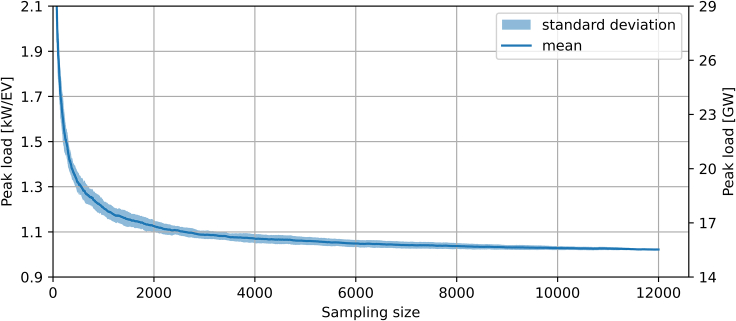
After Diversity Maximum Demand (ADMD) as a function of sample size The profiles’ temporal scope is 3 months. The dark blue line and the light area indicate the mean and the standard error, respectively. The variation results from calculating the ADMDs 50 times with a random order of profiles for every sample size.

As expected, Figure 1 indicates a substantial decrease in fleet peak load when accounting for more profiles. This means that representing a large EV fleet with too few profiles may overestimate the peak. For example, using 100 instead of 10,000 profiles would result in a peak load that is approx. 85% higher. For a fleet of 15 million EVs in Germany in 2030, this overestimation would correspond to more than 13 GW or more than 10% of the currently installed dispatchable generation capacity.^38^ The shape of the mean ADMD is similar to a hockey stick, indicating that marginal accuracy gains decrease with the number of profiles. For example, beyond roughly 5,000 profiles, accounting for an additional 1,000 profiles impacts the fleet peak load by less than 1%, which may be conceived as sufficiently accurate. Although this number may be used as a representativity benchmark, other accuracy thresholds are investigated in Note S2. Hereafter, we will use 12,000 profiles to adequately represent German EVs in 2030.

**Figure 2:**
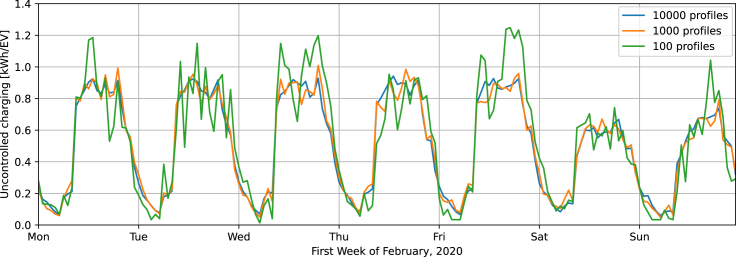
Normalized charging profiles based on 100, 1,000, or 10,000 individual profiles The profiles assume an uncontrolled charging strategy. The peak load corresponding with using 100 is significantly higher than that of the other two profiles.

Figure 2 further illustrates what using 100 or 1,000 instead of 10,000 profiles implies for daily fleet grid demand patterns. The pattern for 10,000 profiles is relatively smooth and may be considered representative of a national EV fleet. By contrast, the consumption pattern for 100 profiles seems unrealistically volatile. For the given time period, the profile corresponding to 1,000 EV profiles is similar to the one corresponding to 10,000 EV profiles. Note that we apply an uncontrolled charging strategy and assume a plug-in rate of 100% in our main scenario. The latter means that, when there is a charging station available, the EV is always connected to it. By contrast, current plug-in rates for public charging are lower, especially during daytime, and this leads to less charging during the day and higher evening peaks in empirically observed charging profiles.^29^^,^^39^ We simulate the effect of reduced plug-in rates for public charging in Note S3, finding a peak load increase of 11%. Yet the power sector implications of this charging behavior are less clear and should be investigated in detail with dedicated energy models. Regarding the question of whether this potential increase in homogeneity accompanied by an increase in peak load reduces the number of profiles necessary for building a representative fleet, we do not find any significant indications.

Our results imply that studies based on too few profiles may substantially overestimate the peak caused by (uncontrolled) EVs. For example, earlier studies that used only between 50 and 200 profiles^23^^,^^24^ found a peak load of up to 1.4 kW/EV, about 40% higher than our estimated peak load of only approx. 1 kW/EV.^23^ Note that our estimated peak is still about twice that of an empirical study performed in the UK in 2017 and 2018^40^; this may be due to our higher assumptions on annual mileage and on the share of public charging, which features higher power ratings (see Muessel et al.^34^ for a more detailed discussion). On the other hand, our assumed plug-in rate of 100% may lead to a relatively low evening peak (see Note S3).

### Why naïve aggregation overestimates the flexibility potential

The previous subsection demonstrated that the number of sampled EV profiles heavily affects the fleet profile’s shape, calling for many individual EV profiles to be considered in energy system models. What adds to the complexity is that each EV could flexibly adjust its charging strategy to help balance the electricity system. However, integrating thousands of individual profiles and their individual flexibility potentials in already complex energy system models is infeasible due to computational constraints. This subsection elaborates on modeling EV-specific flexibility potentials through power and energy constraints and the implications of naïvely aggregating these constraints into one set of constraints for the entire fleet. We do so based on a visualization of flexibility potentials over time and provide equations in the Methods section.

**Figure 3:**
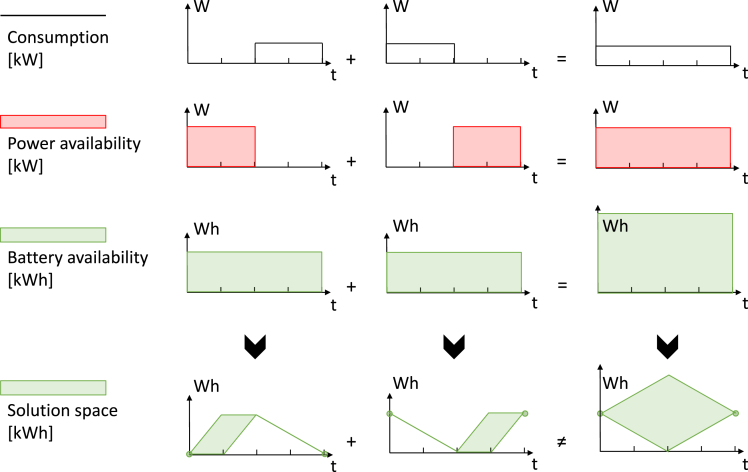
Naïve aggregation of EV flexibility potentials The flexibility potential of an EV is defined by the electricity consumption for driving (first row), the power availability for charging (second row), and the battery availability (third row). The solution space (fourth row) represents the range of feasible battery levels given the constraints from above and assuming fixed initial and final battery levels (green circles in the fourth row). The first two columns depict individual EVs and the third column their naïve aggregation.

Here we define the flexibility of a single EV with three time series, as depicted in the first two columns of Figure 3 for two exemplary EVs. First, the consumption time series defines how much the battery level decreases while driving (first row in Figure 3). This series also implicitly determines to what minimum level the EV must be charged before starting a trip. Second, the power availability time series defines the maximum power for charging the battery (second row). In our example, the available charging power is always positive, i.e., no bidirectional charging is allowed. Third, the battery availability time series captures the usable size of the battery, which limits the maximum battery level (third row). Combined, these three time series determine the solution space for the battery level, i.e., the range of battery levels that is feasible when respecting the power and energy constraints imposed by consumption, power availability, and battery availability. This is depicted in the fourth row of Figure 3, under the assumption that the battery of the EV in the first column is empty whereas that of the EV in the second column is full, both at the beginning and at the end of the depicted period. The size of the solution space can be interpreted as the virtual storage size, i.e., the flexibility potential.

The third column in Figure 3 shows the naïve aggregation of the power and energy constraints for the two exemplary EV profiles in the first two columns. When speaking of the naïve aggregation, we refer to a line-wise addition of the required consumption, the power availability, and the battery availability (rows 1–3). The fourth row in the third column shows the solution space resulting from these aggregated constraints. The size of this solution space exceeds the sum of the individually derived solution spaces in the first two columns. Although this larger solution space respects the naïvely aggregated constraints, some points in the aggregate solution space are infeasible on the level of individual EVs. For example, following the upper boundary of the aggregated solution space would imply charging the vehicle that is connected during this period at full capacity over two time steps. However, the connected vehicle will already be fully charged after one time step, and continuing to charge during the second time step would effectively mean charging the other vehicle while driving, which is impossible. In other words, a naïve aggregation of individual EVs leads to the loss of some information contained in the individual constraints and an overestimation of the fleet’s flexibility potential.

### How the virtual energy storage approach solves the aggregation problem

To avoid the overestimation of the flexibility potential, we propose a novel aggregation approach that is based on modeling flexibility as a virtual energy storage. The approach is based on the idea that the flexible charging of an electric vehicle can be decomposed into an inflexible reference charging strategy and the flexible deviation from that reference. Charging more compared with the reference can be associated with charging the virtual energy storage, whereas charging comparatively less results in discharging the virtual energy storage.^41^^,^^42^ Here, we consider an uncontrolled charging strategy as the inflexible reference, but other reference strategies are possible. Referring back to Figure 3, an uncontrolled charging strategy means following the upper boundary lines of the individual solution spaces (fourth row).

**Figure 4:**
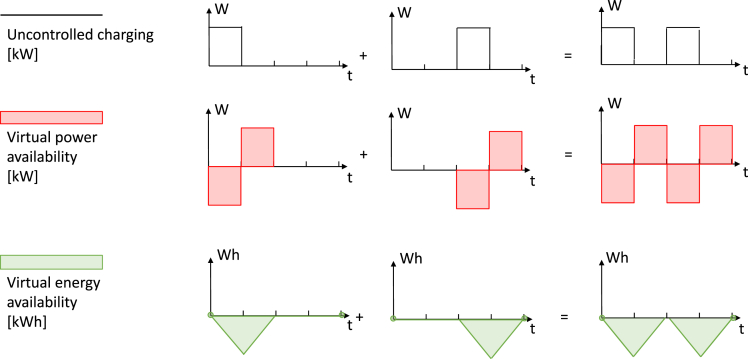
Virtual aggregation of EV flexibility potentials The virtual energy storage approach is defined by an inflexible reference consumption and the potential to deviate from this reference. In our case, we define uncontrolled charging as the reference (first row). The virtual power availability characterizes the potential for deviating from this reference in power terms (second row). The virtual energy availability characterizes the potential for deviating from the reference in energy terms and coincides with the solution space for the virtual energy storage level (third row), assuming fixed initial and final storage levels (green circles in the third row). The first two columns depict the virtual energy storages corresponding to individual EVs, and the third column is the aggregation of the virtual energy storages.

The results of applying the virtual energy storage approach to the exemplary electric vehicles from Figure 3 are illustrated in the first two columns of Figure 4. Instead of the consumption from the battery for driving, the virtual energy storage approach considers the inflexible reference consumption from the electricity grid as an exogenous consumption time series, which follows an uncontrolled charging strategy in our case (first row). On that basis, the available power for charging and discharging the virtual energy storage can be derived as the maximum deviation from this (uncontrolled) reference charging strategy (second row). Note that, in our example, the negative charging power availability of the virtual energy storage does not imply the possibility of feeding electricity back into the grid (bidirectional charging). Instead, more generally, it reflects the ability to charge less than in the reference. Finally, the available energy capacity of the virtual energy storage is the maximum deviation of the EV’s battery level from the battery level in the uncontrolled reference (third row). It is important that one accounts for all possible energy and power constraints imposed on the EV when deriving this maximum deviation. As a result, the available virtual energy storage capacity coincides with the solution space for the virtual energy storage level. Hence, there is no need for a fourth row in Figure 4. Note that, in our example, the available virtual energy storage capacity is always negative; this is because we use uncontrolled charging as a reference consumption, and a deviation from this reference would always lower the battery level. (For a detailed description of the virtual energy storage approach, see STAR methods.)

The third column in Figure 4 shows the aggregation of the two EV-derived virtual energy storages in the first two columns. Analogously to the naïve aggregation, we perform a line-wise aggregation of the virtual constraints. However, in contrast to the naïve aggregation, the resulting solution space is accurate in energy terms. It does not lead to an overestimation of the solution space because the available energy capacity of the virtual storage *is* the solution space. This aggregation can be easily scaled up to thousands of EV profiles, as we show in the following section, and implemented into energy system models for a more accurate representation of EV flexibility.

### An application of the virtual energy storage approach to Germany 2030

To demonstrate the benefits of our approach, we apply it to the case of Germany in 2030. To this end, we use the 12,000 individual profiles from above and apply the described virtual energy storage approach to each profile. To derive each individual flexibility potential, we make assumptions on people’s behavior and their willingness to provide flexibility services: we assume that people are willing to provide flexibility at nonpublic charging stations via unidirectional smart charging, under the condition that the battery level reaches the level corresponding to uncontrolled charging before the next trip starts. In other words, we neither consider flexibility at public charging stations, bidirectional charging, nor the potential to charge less before a drive that does not require a fully charged battery. We also take the mobility behavior as fully exogenous, i.e., we do not consider the potential to postpone (or prepone) a ride to provide flexibility to the power system. Note that these assumptions are made for a largely conservative estimate of flexibility potentials. In Note S4 and S5 we provide sensitivity analyses in which we consider the additional flexibility potential from public charging and an even more conservative flexibility scenario where battery levels are low. After determining the individual virtual energy storages, we aggregate them on the fleet level and scale the aggregated constraints to the expected fleet size of 15 million EVs. Note that we aggregate EV flexibility across Germany and discuss the possibility to extend our approach to handle spatial information in Note S7. Furthermore, we provide more details on the assumptions and the input data for the case study in Note S1.

**Figure 5:**
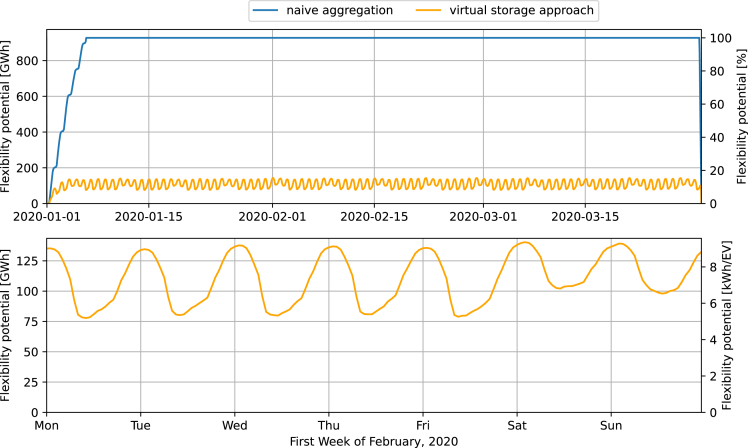
Comparison of the solution spaces with naïve and virtual aggregation The upper plot indicates the significant size of the overestimation when comparing the flexibility potential associated with the naïve aggregation and that associated with the virtual energy storage approach. The explicit constraints for initial and final battery levels strongly determine the battery level and virtual energy storage constraints around the first. The lower plot shows in more detail the diurnal characteristics of the virtual energy storage level only—and last-time step—n = 12,000 quarterly profiles.

Figure 5 displays the resulting flexibility potentials of the EV fleet in terms of the absolute sizes of the naïvely and virtually aggregated solution spaces (see STAR methods for details on the calculations). The lower plot shows that the size of the virtual energy storage size does not exceed 125 GWh, which is only 13% of the maximum size of the solution space resulting from naïve aggregation. The latter (927 GWh) almost coincides with the aggregated battery capacity of 930 GWh (15 million EVs ∗ 62 kWh of battery capacity). On average, the naïve solution space is about 10 times larger than the virtual energy storage solution. Note that the first- and last-time steps are fundamentally different from the others because of the explicit battery level constraints (recall the green circles in Figure 3); these determine that there is no potential to deviate from an uncontrolled charging strategy, regardless of the aggregation method.

Although the naïve aggregation leads to an almost constant flexibility potential of 900 GWh, the availability of virtual energy storage capacity fluctuates starkly between 69 and 125 GWh according to the diurnal driving consumption patterns (lower plot in Figure 5). Between 12 p.m. and approx. 3 a.m., the flexibility potential associated with the virtual energy storage approach is the highest, which seems plausible because most EVs are not used during that time. On the other hand, when most EVs are used during the daytime, the flexibility potential is the smallest. Furthermore, the flexibility potential during weekends is significantly higher as fewer people commute to their workplaces. By contrast, the solution space corresponding to naïve aggregation shows an almost completely flat line, apart from the initial and final time steps. This corresponds with the virtual energy storage accounting for individual flexibility constraints, e.g., that the EV must reach the battery level corresponding to uncontrolled charging before starting a trip. Hence, the individual trips impact the size of the solution space.

## Discussion

### Interpretation of the results

Regarding the accurate representation of EVs’ volatility in energy system models, we find that multiple thousands of individual profiles are needed to generate accurate results, whereas earlier studies^23^^,^^24^ often used below 200 profiles. Using 100 profiles in comparison to using 10,000 profiles leads to a peak load increase of about 85% (approx. 1.9 kW/EV vs. 1 kW/EV). This deviation is quite significant when assuming EV fleet sizes of multiple million EVs. For example, the projected 15 million EVs in Germany in 2030 imply a deviation of 13 GW, which is about 17% of the currently installed dispatchable national generation capacity. Note that we assume a plug-in rate of 100% and an uncontrolled charging strategy. In fact, plug-in rates may be lower during the day, which would shift some consumption to the evening hours and increase the overall peak at that time. Furthermore, lower plug-in rates during the day lead to more homogeneous charging profiles, which can be more accurately represented by fewer profiles (see Note S3). Another critical assumption of our study concerns mobility behavior, which we base on current mobility statistics. However, the development of drive trains may influence mobility behavior by 2030.^43^ Public transport, shared mobility services, autonomous driving, and events such as pandemics might significantly change or disrupt mobility patterns. If this would make driving patterns more heterogeneous, one would need to account for more profiles than in our case.

Regarding the accurate representation of flexibility, we find that a naïve aggregation of individual flexibility potentials leads to a significant overestimation of the fleet’s flexibility. We introduce a novel virtual energy storage approach for a mathematically accurate aggregation of individual flexibilities and find a fleet flexibility potential that is 10 times smaller than with naïve aggregation. With that in mind, values about the flexibility potential in the literature should be taken with a grain of salt as they could, without additional assumptions, be tremendously overestimated. However, current energy system studies on EV flexibility potential already assume the potential to be significantly below the aggregated battery sizes and hence below the result of naïve aggregation. How much lower was chosen by industry experts and heuristics.^4^^,^^44^^,^^45^^,^^46^ For example, a recent study of the German energy system assumes a constantly available flexibility of 5.7 kWh/EV.^47^ Although this is similar to our estimated average of 6.2 kWh/EV, we see significant diurnal changes in the EVs’ average flexibility potential (between 4.5 and 8.3 kWh/EV). Note that Luderer et al.^47^ partly allows for bidirectional charging of a fraction of the EVs, but it remains unclear how much this impacts the results.

Our case study for Germany in 2030 with 15 million EVs yields a 93 GWh average potential to shift electricity over time through smart EV charging. To put this into perspective, it can be compared with current national installed capacities for large-scale batteries (750 MWh) or pumped hydro storage power plants (39 GWh).^48^^,^^49^ Against this background, the 93 GWh represents a flexibility potential that can be significant for the overall system stability in decarbonized energy systems. To give a rough indication of the associated potential for system cost savings, we assume that the EV’s flexibility substitutes proportionately for stationary batteries at specific costs of 135€–450€/kWh,^50^ and this leads to estimated cost savings of about 27 billion €. However, an accurate estimation of the reduction in systems costs can only be derived by integrating the EVs’ flexibility potential into an energy system model.

We made three critical behavioral assumptions regarding flexibility potentials. First, we assume that people are willing to provide flexibility conditional on the EV reaching the same level as with uncontrolled charging before starting a trip. In fact, previous studies have reported that the majority of people are willing to provide flexibility under this condition.^51^^,^^52^^,^^53^ Furthermore, as there is some degree of range anxiety, which is associated with the battery, we provide a sensitivity analysis in Note S5 on the battery levels that should be avoided to undergo. We find that avoiding to undergo a certain battery has a limited effect on the average solution space size (below 2%). Second, we assume unidirectional charging and, thus, do not account for vehicle-to-grid potentials. Third, we assume a plug-in rate of 100%. However, in reality, people might be less likely to plug in their EV in as soon as possible; this is especially true for public charging stations, where people mostly charge when and for the duration they have to due to their mobility requirements.^54^^,^^55^ Considering the significant role of public charging (high power ratings), and the characteristic situation that people are less likely to plug in their EV unless they require the charging process, we assume no flexibility potential from public charging stations. Hence, we might overestimate the flexibility potential for nonpublic charging stations with the plug-in rate of 100%, while we ensure a conservative estimate for public charging stations with the assumption of no associated flexibility potential. In Note S4 we show that the flexibility potential could increase by 16% when allowing for full flexibility at public charging stations.

### Limitations of the study

There are three noteworthy limitations to our aggregation approach: it requires the mobility behavior to be exogenous, it neglects intertemporal dependencies in power availability, and it takes an exogenously defined battery level at the start and end of the modeled period as an input. See Note S6 for a detailed discussion of these limitations.

### Conclusions

Modeling EVs’ net impact on the energy system in the next decade is fundamental for policymaking and infrastructure planning. Current methods use small samples of charging profiles or following naïive aggregation methods. This results in a significant overestimation of the volatility of EVs and their flexibility potential. This article demonstrates how to determine a representative sample size for modeling mobility behavior on a system level, using peak load as a comparison metric. Furthermore, it develops and applies a novel aggregation method with the potential to advance the accuracy of current aggregation methods. As a unique feature, our approach can easily be implemented into energy system models for an endogenous determination of prices and dispatch.

This novel aggregation method, which we refer to as a virtual energy storage approach, is the main methodical contribution of this article. The general idea is that the EVs’ flexibility potential is modeled as the deviation from an inflexible reference charging strategy while respecting its primary purpose: mobility. The flexibility potentials are characterized in terms of power and energy constraints and can be aggregated without accuracy losses, in contrast to the naïve aggregation of individual constraints without applying the virtual energy storage approach. Note that with this approach the runtime increases only linearly with the number of time steps and remains constant when increasing the number of EVs modeled. Hence, the virtual energy storage approach yields the potential for a run-time efficient and accurate optimization based on the aggregated constraints.

We applied our method to a case study with 15 million EVs in Germany in 2030. The results show that, in comparison to an uncontrolled charging strategy, adapted charging can provide a sizable average flexibility potential of 93 GWh (6.2 kWh/EV). Furthermore, the naïve aggregation significantly overestimates the system requirements 10-fold in terms of peak load from electric mobility.

Further research may build on this work. Regarding model development, accounting for bidirectional charging is an important next step. Our method provides a foundation for assessing the flexibility potential of EVs on a system level. To assess EVs' flexibility potential in the context of the energy system, the aggregated virtual energy storage profile could be integrated into a power market model. Besides the EV-specific application, this method holds the potential to be applied to a range of other flexible demand-side applications, such as electric heat pumps.

## STAR★Methods

### Key resources table

**Table:** 

REAGENT or RESOURCE	SOURCE	IDENTIFIER
**Software and algorithms**		

Python version 3.7.10	Python Software Foundation	https://www.python.org
Python package emobpy version 0.6.2	Gaete et al.^23^	https://doi.org/10.1038/s41597-021-00932-9
Virtual Storage Algorithm		https://github.com/jmuessel/VirtualStorageEV

### Resource availability

#### Lead contact

Further information for resources and materials should be directed to and will be fulfilled by the lead contact, Jarusch Muessel (mailto:Jarusch.muessel@pik-potsdam.de).

#### Materials availability

This study did not generate new unique materials

### Method details

This section presents the quantitative modeling of EVs and their flexibility potential. Firstly, we introduce the necessary input data and equations for modeling individual EVs. This part does not claim to contribute any novel modeling approaches and could be extended in various ways as our method is agnostic to the addition of constraints. Afterward, the aggregation of flexibility potentials based on a naive approach and based on the virtual energy storage transformation is presented in turn. Sensitivity analyses for different parameters are provided in Notes S4 and S5.

#### Modeling individual EVs

To model the solution space of an EV’s battery level, we rely on three exogenous time series: the electricity consumption for driving, $consumption_{i,t}$, the power availability for charging, $charge_{i,t}^{\max}$ (both in terms of power, e.g., kW), and the battery availability (in terms of energy, e.g., kWh).

Combined, Equations 1, 2, 3, and 4 determine the solution space for the decision variables ${LEVEL}_{i,t}$ (in terms of energy, e.g., kWh), which captures the energy level in the battery, and ${CHARGE}_{i,t}$ (in terms of power, e.g., kW). The solution space includes all feasible battery level profiles that respect the power and energy constraints imposed by consumption, power availability, and battery availability (in terms of energy, e.g., kWh). This range of battery levels determines the potential to shift energy. The size is given in terms of energy.

The battery level represents the charged energy available to a vehicle at time *t* in terms of energy in kWh. According to Equation 1 it is recursively defined and impacted by the decision to charge and the exogenous consumption for driving. The consumption time series is expressed as constant power in kW while the battery level is given as energy in kWh for every time step. Assuming a constant consumption per time step and a time step of 1 h, the energy consumption of a trip is simply the sum of power consumption over the time steps when the EV drives. The energy consumption for driving implicitly determines to what minimum level the EV must be charged before starting a trip. This is ensured by the recursive battery level definition in Equation 1. Note that the battery level is determined exogenously in the initial and final time step. We assume in both time steps full battery capacity. The battery availability time series captures the usable size of the battery. It can be freely chosen but must not exceed the battery capacity and remain non-negative, cf. Equation 2.

The power station availability determines at which power rating the EV can be charged. In our case study, we account for positive charging power availability, without the possibility for bidirectional charging. Therefore, $charge_{i,t}^{\min}$ is zero in Equation 3

∀[i,t∈I,T]

(Equation 1) $$LEVEL_{i,t}=LEVEL_{i,t-1}+\left(CHARGE_{i,t}-consumption_{i,t}\right)*\Delta t$$

(Equation 2) $$level_{i,t}^{\min}\leq LEVEL_{i,t}\leq level_{i,t}^{\max}$$

(Equation 3) $${charge}_{i,t}^{\min}\leq {CHARGE}_{i,t}\leq {charge}_{i,t}^{\max}$$

In our main scenario, we do not allow for flexibility from public charging This is implemented in Equation 4, which implies that the EV must follow an uncontrolled charging strategy when connected to a public charging station, i.e., for the subset from *T, T*_*public*_. In Note S4, we describe the role of public charging in more detail and do a sensitivity analysis in which we allow for flexibility at public charging stations.

$$\forall \left[i,t_{pub}\in I,T_{public}\right]$$

(Equation 4) $${LEVEL}_{i,t_{pub}}=level_{i,t_{pub}}^{uncontrolled}$$

Furthermore, Equation 5 ensures that, before starting the next trip, the EV reaches the level that corresponds with uncontrolled charging in the respective time step. Therefore, additional subsets are used that include the departure times for each vehicle: $T_{i,departures}$. This is a crucial model extension as peoples’ willingness to provide flexibility heavily depends on the battery level the EV reaches before starting the next trip.^56^ For a discussion on behavioral modeling aspects see Notes S4 and S5.

$$\forall \left[i,t_{i,dep}\in I,T_{i,departures}\right]$$

(Equation 5) $$LEVEL_{i,t_{dep}-1}=level_{i,t_{i,dep}-1}^{uncontrolled}$$

#### Naive aggregation

Equations 6, 7, and 8 show the fleet constraints when following the naive aggregation approach. This implies that for each constraint we combine all sub-constraints for each EV into a fleet constraint.

∀t∈T

(Equation 6) $$LEVEL_{t}^{fleet}=LEVEL_{t-1}^{fleet}+\left(CHARGE_{t}^{fleet}-\sum_{i=1}^{I}consumption_{i,t}\right)*\Delta t$$

(Equation 7) $$\sum_{i=1}^{I}{level}_{i,t}^{\min}\leq LEVEL_{t}^{fleet}\leq \sum_{i=1}^{I}{level}_{i,t}^{\max}$$

(Equation 8) $$\sum_{i=1}^{I}{charge}_{i,t}^{\min}\leq CHARGE_{t}^{fleet}\leq \sum_{i=1}^{I}{charge}_{i,t}^{\max}$$

From this aggregation, we lose information, e.g., the constraint to reach a battery level that equals the energy amount the EV would have reached in the uncontrolled charging case, one time step before starting a trip, Equation 4. The same holds for the information about public charging. We could aggregate Equations 4 and 5 for all EVs i in *I*, in the same way, we aggregate Equations 1, 2, and 3. Thereby, we would lose information about the state in which an EV is with its respective properties: driving, parking (with grid connection and with how many steps until the next trip), and charging. This information loss leads to an overestimation of the flexibility potential.

#### Virtual energy storage aggregation

The virtual energy storage capacity profile builds on a battery level’s solution space, respectively on Equations 1, 2, 3, 4, and 5. The main idea is now to decompose the flexible charging of an EV into an inflexible reference charging strategy and the flexible deviation from that reference. Charging more compared to the reference can be associated with charging the virtual energy storage, and charging comparatively less results in discharging it.^41^^,^^42^ Table Effect of different charging strategies in the virtual energy storage level. Summarizes the effects of different deviations on the virtual energy storage level. We consider an uncontrolled charging strategy (charging as early and much as possible) as the inflexible reference, but other reference strategies are possible as well. Referring to the definition of the objective function in Equation 9, an uncontrolled charging strategy would imply maximizing it. To determine the virtual energy storage capacity, the difference between the furthest deviation from charging as early as possible is calculated for every time step: charging as late as possible. Minimizing the sum of batteries, Equation 9, subject to Equations 1, 2, 3, 4, and 5 leads to this profile, while maximizing leads to the battery level profile corresponding with uncontrolled charging.

∀i∈I

(Equation 9) $$\text{Objective}\;\text{function}=\sum_{t=0}^{T}{\text{LEVEL}}_{t,i}$$

**Table:** Table Effect of different charging strategies in the virtual energy storage level.

Charging strategy	Virtual energy storage level
Uncontrolled:	Neutral
Smart: Postponing uncontrolled charging	Initially, charging less than uncontrolled charging: discharging the virtual energy storage Later, charging more than uncontrolled charging: charging the virtual energy storage
Bidirectional: smart charging with the possibility to discharge the EV to the grid	Initially, charging less than uncontrolled charging or even discharging the battery: discharging the virtual energy storage Later, charging more than uncontrolled charging: charging the virtual energy storage

In general: charging less than in the uncontrolled case leads to discharging the virtual energy storage.

Note that for *t = 0* and *t = T* the virtual energy storage capacity is defined explicitly. Thus, even when applying adapted charging strategies, it is impossible to divert from this level. This significantly impacts the potential virtual energy storage levels for time steps close to the beginning and end of the considered time horizon.

After maximizing and minimizing the objective function in Equation 9 according to Equations 1, 2, 3, 4, and 5, we subtract the late-charging profile (maximization) from the early charging profile (minimization) for an EV profile *i*, Equation 10 to determine the lower bound of the virtual energy storage profile. The upper bound of the virtual energy storage profile corresponds with the minimal deviation from the reference profile, which is 0 for every time step, Equation 11. Notably, these virtual energy constraints already incorporate the relevant information of each individual EV’s energy requirements to allow for the exogenous mobility demand.

∀[i,t∈I,T]

(Equation 10) $${level}_{i,t}^{vs,\max}=0$$

(Equation 11) $${level}_{i,t}^{vs,\min}={level}_{i,t}^{late}-{level}_{i,t}^{uncontrolled}$$

Analogously to the naive aggregation, Equations 6, 7, and 8, we perform aggregation for every set of constraints: power availability, available capacity of the virtual energy storage, Equations 12, 13, and 14. The power constraint is determined by the physical charging potentials and the potential to deviate from an uncontrolled charging strategy, Equation 14. Note that Equation 12 does not include the exogenous time series for uncontrolled charging, which must be considered separately. Furthermore, note that the virtual storage level and charging can now generally take postive and negative values.

∀t∈I

(Equation 12) $${LEVEL}_{t}^{vs,fleet}={LEVEL}_{t}^{vs,fleet}+{\Delta t*CHARGE}_{t}^{vs,fleet}$$

(Equation 13) $$\sum_{i=1}^{I}{level}_{i,t}^{vs,\min}\leq {LEVEL}_{t}^{vs,fleet}\leq \sum_{i=1}^{I}{level}_{i,t}^{vs,\max}$$

(Equation 14) $$\sum_{i=1}^{I}{charge}_{i,t}^{\min}+{charge}_{i,t}^{uncontrolled}\leq {CHARGE}_{t}^{vs,fleet}\leq \sum_{i=1}^{I}{charge}_{i,t}^{\max}-{charge}_{i,t}^{uncontrolled}$$

## Data Availability

• Data reported in this paper will be shared by the lead contact upon request. • All original code has been deposited on Github and is publicly available as of the date of publication. URLs and DOIs are listed in the key resources table. • Any additional information required to reanalyze the data reported in this paper is available from the lead contact upon request.

## Supplemental information

Document S1. Figures S1–S4, Table S1, and Notes S1–S7
