# Supplementary material for: Accurate and scalable representation of electric vehicles in energy system models: A virtual storage-based aggregation approach
Source: iScience. 2023 Sep 1;26(10):107816. doi: 10.1016/j.isci.2023.107816 (PMC10510052; doi:10.1016/j.isci.2023.107816)
Supplement: Document S1. Figures S1–S4, Table S1, and Notes S1–S7 [file mmc1.pdf]

iScience, Volume *26*

Supplemental information

Accurate and scalable representation of electric vehicles in energy system models: A virtual storage-based aggregation approach

Jarusch Muessel, Oliver Ruhnau, and Reinhard Madlener

SUPPLEMENTAL INFORMATION (SI)

This document provides further methodical details, sensitivity analyses, and additional discussions of the assumptions made in the main text. First, we briefly describe the tool behind the input data generation. Second, the After Diversity Maximum Demand concept is explained in detail, and additional simulation runs are represented. Third, the effect of reduced plug-in rates during the day on peak load is analyzed. Fourth, we perform sensitivity analyses on flexibility-determining parameters. Lastly, the limitations of our modeling are discussed in some detail.

NOTE S1: PROFILE SIMULATION - A BRIEF TOOL DESCRIPTION AND DISCUSSION OF INPUT DATA ASSUMPTIONS

For the profile simulation, we choose *emobpy*, a stochastic profile generator that is based on national mobility statistics.[1] We chose *emobpy* because it is a well-documented and validated open-source tool with an active user community. With *emobpy* we sample profiles to represent 15 million EVs as this is an established projection until 2030.[2] The profile generation process follows the order of sampling first, a mobility, second, a consumption, and third, a grid availability profile.[3] For a detailed analysis of the sampled fleet properties see Muessel et al.[4] and the tool description itself.[3] *emobpy*'s built-in plausibility check ensures that the different time series and assumptions are consistent. Most importantly, it ensures that the sampled mobility profile is feasible considering the availability of charging stations and the battery size. *emobpy* samples for every time-step, location, and state (driving, parking) the power availability. Power availability means that the EV has access to a charging station with a specific power rating and that it is plugged into it. The power availability fundamentally impacts the number and length of trips that are possible in the future.

In addition to the built-in mobility statistics, *emobpy* uses physical vehicle properties and customizable assumptions, e.g., on infrastructure availability as an input. Table S1 provides an overview of power availability probabilities, which we set explicitly in accordance with a national mobility survey and expert guesses.[1,3] The power rating at each location is: public: 22 kW, workplace: 11 kW, home: 3.7 kW. Additional parameters comprise the EV model chosen, which influences the battery size and the specific consumption. We account for 10 different EV models, based on EV sales in Germany in 2021[5] with a focus on EV models with a large battery capacity, as by 2030 significant technological progress toward larger battery capacities can be estimated. We compute an average battery capacity of 62 kWh.

Table S1: Probability parameters for the station distribution along different locations. Related to Figure 2

Location/states	Probability for power availability
Public, errands, shopping, escort, leisure	0.5
None	0
Home	1
Workplace	1
Driving	0.01*

*To allow for long trips there is a very low probability for public fast charging while the EV is in the state 'driving': {'none': 0.99, 'fast75 kW': 0.005, 'fast150 kW': 0.005}

Most of the time, EVs are parked at different locations, which corresponds well with values from the literature for idle times of above 90%.[3] During the night (between 11:00 PM and 5:00 AM), on average more than 97% of the fleet is at home. diurnal pattern of average fleet consumption corresponds with those for mobility. Note that there is some seasonality in consumption (varying specific consumption), which is modeled by *emobpy* as a function of the calendar date to account for lower (average) ambient temperatures in winter. This means that additional energy is consumed for heating the EV interior space.

The fleet's average power availability is about 10 kW, with some variation throughout each day. This corresponds with varying availability and ratings of charging stations: At home, the availability is usually higher, but the power rating lower than that at public stations, especially when fast charging stations are considered.

emobpy accounts for two different driver types: commuters (62%: 22% part-time, 78% full-time) and non-commuters (38%).[3] Drivers with a service trip destination, e.g., taxi drivers, are excluded according to.[3] It is assumed that individuals with access to a vehicle carry out all their trips with the same vehicle. Information on the 10 EV profiles considered can be found in Table S1 (see Note S2). To summarize the assumptions on grid availability, *emobpy* assumes significantly higher availability for charging stations at home (3.7 kW) and at the workplace (11 kW), compared to public (22 kW) and fast charging stations (50 to 150 kW). The efficiency for charging is assumed to be 90%.[6,7]

The profile sampling relies on national mobility statistics pre-2019 and expert assumptions.[1] Meanwhile, the case study's assumption on EV diffusion reflects projections for 2030, and with the development of drive trains, mobility behavior will probably also change. Public transport shared mobility services, (potentially) autonomous driving, and events such as pandemics can significantly change mobility patterns.[8] This could change the number of profiles necessary for modeling a representative fleet and an EV's average flexibility potential.[9]

The sampling tool makes several simplifying assumptions throughout the sampling process, see Muessel et al.[4] for additional details. Note that *emobpy* uses only abstract locations and no spatial resolution. These locations are workplace, shopping, errands, escort, leisure, and home.

When modeling actual grid transmission, this would be a significant drawback and EV profiles that comprise ZIP codes or GPS data would be required.

NOTE S2. AFTER DIVERSITY MAXIMUM DEMAND (ADMD)

To determine a representative fleet size we use the concept of After Diversity Maximum Demand (ADMD) or, in the case of EVs, peak load.[10] According to Eq. (S1), we calculate the peak load or ADMD by cumulating the fleet grid demand for each time step and determining the maximum of this resulting time series.

$$ADMD = max_{t \in T} \left(\sum_{n=1}^{N} gridDemand_{t,n} \right) \tag{S1}$$

To determine a representative number of profiles, we analyze the peak load or ADMD development that corresponds with increasing the number of sampled EV profiles. The sample starts with 10 EVs and increases to 12,000 profiles. Our input profiles are sampled based on mobility statistics.[1] Therefore, the term "representative" would imply that the fleet profile's behavior converges to that of the underlying probability distribution. The description of all data sources, and especially the sampling of the profiles, can be found in Note S3.

The peak load behavior depends on the overall sample size and changes with an increasing number of profiles. Still, , in the main text, shows a constantly decreasing marginal information gain from using additional profiles. To determine a specific representative fleet size we calculated the relative difference in ADMD when considering additional profiles. Logically, this difference decreases with increasing fleet size. Figure S1 indicates that beyond roughly 5,000 profiles, accounting for an additional 1,000 profiles impacts the fleet peak load by less than 1%. This criterion is somewhat arbitrary but can be used as a benchmark.

Note that when changing the order in our dataset, we would have gotten an overall similar but still different result. To account for this, we performed our ADMD calculation 50 times and ordered the dataset randomly every time. We performed 50 iterations as this seemed reasonable considering the increase in runtime and additional information. The line plot corresponds with the mean ADMD of all iterations.

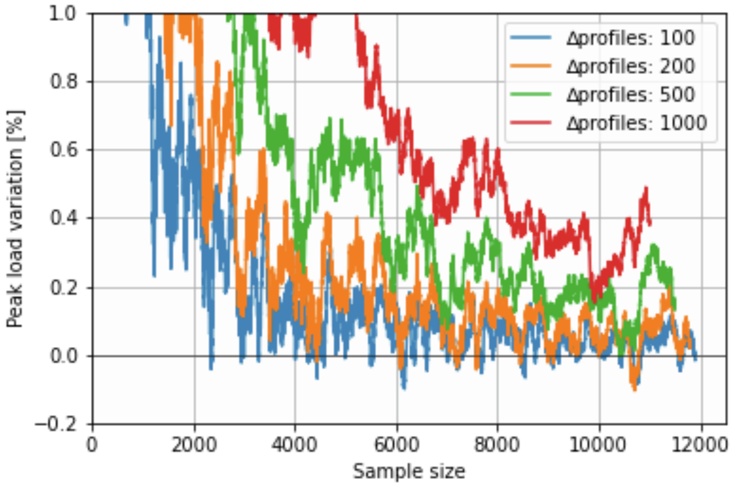

Figure S1: Relative ADMD or peak load development. Related to Figure 1
ADMD variation when using additional profiles. The different line plots result from varying the profile step size for calculating the differences between using a step size with more profiles: for the blue line we compare each fleet size-dependent peak load with that corresponding to a fleet size with additional 100 profiles, whereas the orange line indicates the same calculation but with 200 additional profiles (green: additional 500 profiles, red: additional 1,000 profiles). Hence the blue line has values until the 11,900th profile, whereas the red line has only values until the 11,000th profile. The absolute differences were normalized to percentual values. The time scope of the 12,000 profiles is 3 months.

In contrast to Love et al.[11] we determine a representative number for EVs that is about 18 times higher (5,000 vs. 275) than the number for heat pumps following the same method with different representativity criteria. This can partly be explained by the higher volatility associated with EVs which results from very high idle times for EVs (<95%) and shorter charging intervals at stations that have a higher charging capacity (up to 150 kW at fast charging stations).[12] Concluding, we suggest using more than 5,000 EVs in the sample and we apply 12,000 in our case study of the German EV sector.

NOTE S3. SENSITIVITY ON THE VOLATILITY IN DAYTIME CHARGING: CONTINUOUS VS. NIGHT-FOCUSED UNCONTROLLED CHARGING

We assume a plug-in rate of 100%. This means that whenever a charging station is available, we assume that the EV is directly connected to it. In reality, people are currently less likely to plug in their EVs, especially at public charging stations – unless they must because of a low battery level.[13–15] To differentiate between the implications on volatility and flexibility we ran two different sensitivity analyses (see Note S3 and Note S4). We find that a lower plug-in behavior during the day leads to increased peak load but also to lower differences in average peak load for different fleet sizes (ADMD analysis in Note S1 see Note S4). Furthermore, we find that ignoring the flexibility potential at public charging stations does not lead to a significant reduction in the overall flexibility potential.

On average, plug-in rates are lower during the day, partly because the share of public charging is higher during this time. This would postpone some consumption to the evening and increase the overall peak at that time. We simulate the effect of reduced plug-in rates during the day by

applying an uncontrolled charging strategy between 11 PM and 4 AM – during these times, the share of public charging is the lowest and the periods for which the EVs are parked are the longest. Outside this time frame, charging only happens if needed to complete daytime trips. Hence, we use the time of the day as a proxy for plug-in behavior. Figure S2 (related to Figure 2 in the main text) indicates that the global peak load increased by 11%. Between Friday evening and Monday morning, median demand at night-time is relatively low because the vehicles are less used on weekends than on working days. Accordingly, any regulatory measures that shift EV charging to night-time periods would lead to substantially less smooth patterns compared to all-day charging.

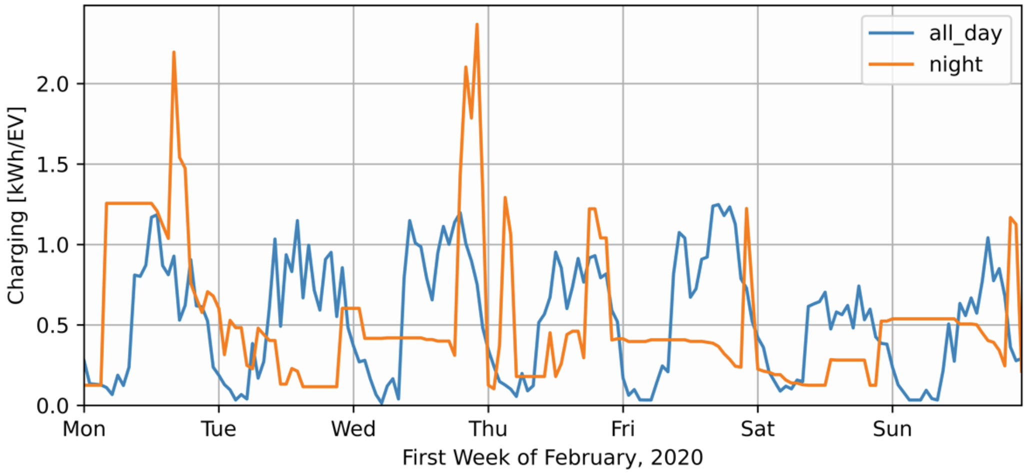

Figure S2: Uncontrolled charging strategies. Related to Figure 1.
The blue shows a plain uncontrolled charging strategy with a plug-in rate of 100%, the orange one a charging strategy that favors night-time charging between 11 PM and 4 AM. We see increased peak loads associated with night-time charging (11%) – n = 100 profiles

NOTE S4. SENSITIVITY ON FLEXIBILITY FROM PUBLIC CHARGING

Literature states a reduced flexibility potential associated with plug-in rates at public charging stations, mainly because of charging location-specific plug-in behavior.[16] Following the proposed approach and not relying on an agent-based model, it is difficult to vary the plug-in behavior, as it was, e.g., by Gschwendtner et al. (2023)[17]. During the aggregation process, we lose the information of a charging event being labeled as public or non-public. Therefore, excluding flexibility potentials from public charging before the aggregation is a pragmatic way to make a conservative estimate of the aggregated flexibility potential. In Figure S3 (related to Figure 5 in the main text) for the blue line, the EV must follow an uncontrolled charging strategy when being connected to a public charging station. The orange line shows the virtual energy storage unit size when allowing charging at public stations to be as flexible as charging at home or at work. With the orange line being approx. 16% larger than the blue line, it becomes clear that the influence of behavioral aspects on demand-side flexibility potentials is important, especially at public charging stations where the power rating is very high. For more details in the mathematical implementation, see Eqs. (4) and (8) in the Method section in the main text.

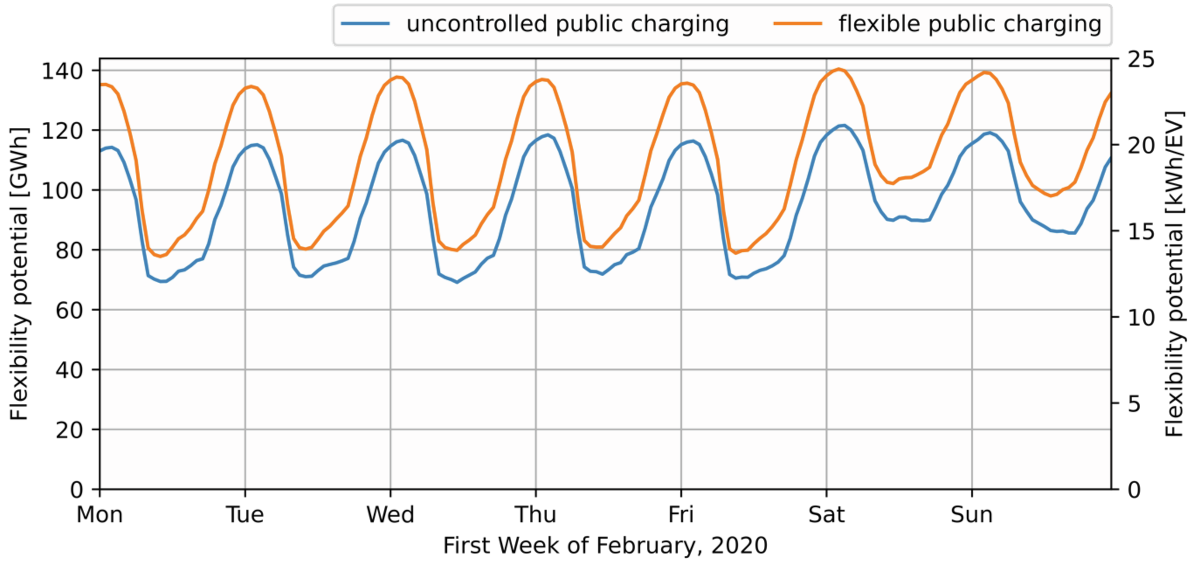

Figure S3: Virtual energy storage unit size. Related to equation 4.

The orange line shows the flexibility potential without the additional flexibility constraint. The blue line corresponds with virtual energy storage unit that includes the constraint that at public charging stations, the EV follows an uncontrolled charging strategy. The impact of public charging stations is significant, as the orange line is 16% higher than the blue one.

NOTE S5. SENSITIVITY ON AVOIDING LOW BATTERY LEVELS

In the main text, we provided the basic formulation for calculating the solution space for charging behavior adaption while, inter alia, accounting for a minimal energy level constraint: $level_t^{min}$. This constraint reflects the minimum level that is required to be as fully charged as possible at the end of any charging event. We now add another constraint (Eq. (S2)) to incentivize the avoidance of low battery levels, $level_t^{\mathrm{low}}$. As this is not always physically possible, we introduce the decision variable $\mathrm{SLACK}^{\mathrm{II}}$. With that, we can model range anxiety or the intention to avoid deep discharging, which could cause a reduction in the battery lifetime.[18,19] In our main scenario, we have set the threshold $level_{i,t}^{\mathrm{low}}$ to zero. Here, we perform a sensitivity analysis, setting it to 10 and 20 percent of the EV's battery capacity.

$$\forall\ [\mathrm{i}, t \in \mathrm{I}, T]$$

$$SLACK_{i,t}^{II} + LEVEL_{i,t} \geq level_t^{\mathrm{min}\_avoid} \tag{S2}$$

We include the decision variable $SLACK_{i,\,t}$ with a negative sign and a high weight, in comparison to the other decision variables (we chose 1,000, following the big M-method), in the objective function, Eq. (S3), to incentivize staying above the level without explicitly constraining it. For the uncontrolled charging strategy, this does not affect the solution for the decision variables $CHARGE_{i,t}$ and $LEVEL_{i,t}$, as low battery levels are avoided in this strategy already. Contrastingly, it shifts the solutions corresponding to charging as late as possible closer to the uncontrolled solution because the optimization criteria here contradict each other: On the one hand, for charging as late as possible, the battery levels are minimized. On the other hand, $\mathrm{M} * SLACK_{i,\,t}$ increases the value for high values for $SLACK_{i,\,t}$. For the aggregated solution space, following

the virtual energy storage unit approach, this means that the input time series for early and late charging are more similar to each other than without Eq. (S2)-(S3).

$\forall\, i \in I$

$$\text{Objective function} = \sum_{t=0}^{T} LEVEL_{i,t} + \mathrm{M} * SLACK_{i,t} \qquad \text{(S3)}$$

Figure S4 (related to Figure 5 in the main text) shows a decrease in the size of solution space with an increase in the additional battery level constraint, but this decrease is very small. The increase in the average solution space size between a threshold of 20% and 0% percent amounts to less than 2%. This is because EV battery levels seldom reach very low levels anyway.

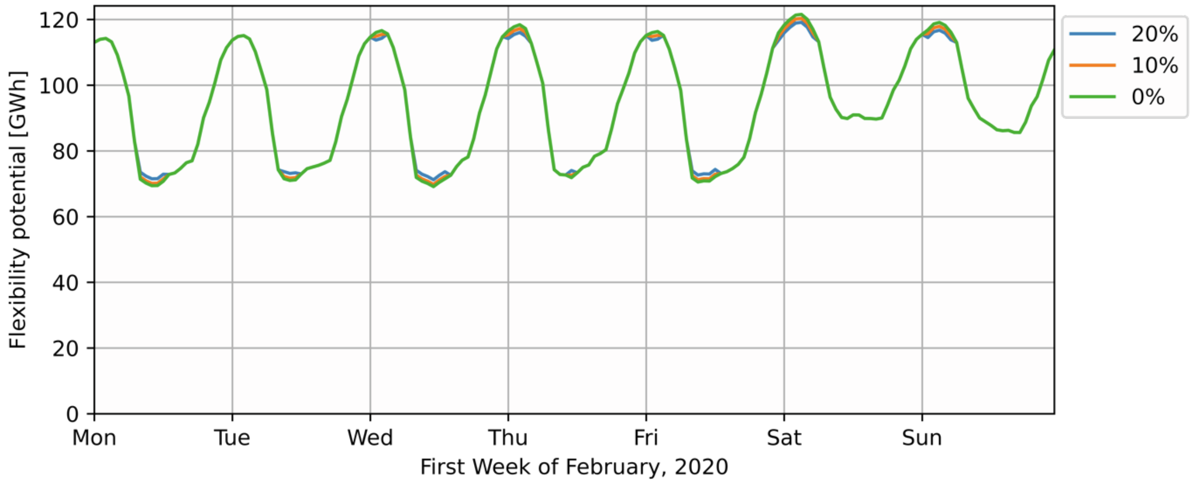

Figure S4: Influence of the constraint on minimal-level avoidance. Related to equation 3.
The blue line shows the flexibility potential corresponding to a minimal battery level of 20% of the battery capacity that should be avoided. The orange and green lines show the flexibility analogously for 10% and 0% of the battery capacity, respectively. The effect of varying this parameter on the average solution space size is below 2%, thus, limited. – n=12,000 weekly profiles

NOTE S6. METHOD LIMITATIONS

This note discusses three limitations to our aggregation approach: the requirement of exogenous assumptions on mobility behavior, the negligence of intertemporal dependencies in power availability, and exogenous inputs on the battery level at the start and end of the modeled period.

First, the requirement of an exogenous assumption on mobility behavior is indeed fundamental for our method. This prevents the consideration of endogenous behavioral changes to provide additional flexibility. However, while some studies do consider such behavioral changes,[17,20] the majority of energy system modeling studies assume mobility behavior to be exogenous.[42] Hence there is are large research communities to which our method is relevant. Not least, our approach with exogenous mobility behavior can provide a lower boundary for EV flexibility in a world where drivers do change their behavior endogenously.

Second, our aggregation approach neglects the intertemporal dependence in power availability. Such intertemporal dependence can occur if an EV is plugged in for ten hours but only needs one hour to be fully charged. Hence the utilization of power from this EV at one hour means

that power from this vehicle becomes unavailable during the other nine hours. On the level of an individual EV, this relationship is captured through the individual energy constraints. On the fleet level, however, the information on the individual relationship gets lost and available power from one vehicle may be used to charge another vehicle faster than it could be charged based on its own power availability. Consequently, while the virtual storage is accurate in energy terms, some solutions that seem feasible based on the aggregated power availability constraints may not be feasibly disaggregated into individual EVs. Intuitively this limitation may be less relevant if EVs are homogenous in terms of their energy-to-power ratio and become significant when heterogeneity increases. A remedy for this limitation may be to aggregate only flexibility potentials with similar energy-to-power-ratios (e.g., separating slow and fast charging). Further research may investigate the relevance of this limitation as well as approaches to enhance the representation of aggregated power availability.

Third, when introducing our approach, we explicitly define the battery level (and hence the virtual energy storage level) for $t = 0$ and $t = T$. Thus, adapted charging strategies cannot divert from this level. This significantly impacts the shape of the virtual energy storage unit solution space for time steps close to the beginning and the end of the considered time horizon (see Figure 5 in the main text). This phenomenon is relevant for energy system models that rely on representative period. If the representative period is rather small, this, so called, convergence problem or time series problem becomes especially critical because it is then represented in every period modeled.[38] A solution to this could lie in the endogenization of the start and end constraints and constraining them to equal each other.

NOTE S7. INTRODUCTION OF SPATIALITY

Due to a lack of input data, we model Germany as one node in our case study (see the beginning of Note S4 for *emobpy's* limitations). However, our approach can be used for a spatially resolved aggregation, e.g., per grid node. Here, we assume exogenous mobility behavior and force the battery level to reach the level it would have reached in the reference case at the end of each charging session. As a result, each charging session can be modeled independent from the other sessions. This makes a spatially resolved aggregation particularly easy, as each charging session can be unambiguously assigned to one of the spatial nodes as soon as spatial information for each session is available. Further research may investigate possibilities of using the virtual storage approach for modeling spatial flexibility, i.e., shifting electricity demand from one node to another.

SUPPLEMENTAL REFERENCES

[S1] Nobis, C., and Kuhnimhof, T. (2018). Mobilität in Deutschland - Ergebnisbericht. http://www.mobilitaet-in-deutschland.de/pdf/MiD2017_Ergebnisbericht.pdf.

[S2] Open Energy Tracker (2022). Elektromobilitätszahlen. https://openenergytracker.org/docs/germany/emobility/.

[S3] Gaete-Morales, C., Kramer, H., Schill, W.-P., and Zerrahn, A. (2021). An open tool for creating battery-electric vehicle time series from empirical data, emobpy. Sci. Data *8*, 152. 10.1038/s41597-021-00932-9.

[S4] Muessel, J., Ruhnau,

O., and Madlener, R. (2023). Simulating charging behavior of electric vehicles: review and comparison with empirical data. In 2023 19th International Conference on the European Energy Market (EEM) (IEEE), pp. 1–7. 10.1109/EEM58374.2023.10161947.

[S5] Car Sales Statistics (2021). EV Sales Shares 2020. https://www.best-selling-cars.com/germany/2021-full-year-germany-best-selling-electric-cars-by-brand-and-model/.

[S6] Chen, X., Zhang, H., Xu, Z., Nielsen, C.P., McElroy, M.B., and Lv, J. (2018). Impacts of fleet types and charging modes for electric vehicles on emissions under different penetrations of wind power. Nat. Energy *3*, 413–421. 10.1038/s41560-018-0133-0.

[S7] Taljegard, M., Walter, V., Göransson, L., Odenberger, M., and Johnsson, F. (2019). Impact of electric vehicles on the cost-competitiveness of generation and storage technologies in the electricity system. Environ. Res. Lett. *14*, 124087. 10.1088/1748-9326/ab5e6b.

[S8] Schlund, J. (2021). Electric Vehicle Charging Flexibility for Ancillary Services in the German Electrical Power System. https://opus4.kobv.de/opus4-fau/frontdoor/index/index/year/2021/docId/17600.

[S19] Krauss, K. (2022). Shared Mobility in MaaS Systems: A Supply and Demand Perspective. http://hdl.handle.net/20.500.11850/594727.

[S10] Mingyang Sun, Konstantelos, I., and Strbac, G. (2016). Analysis of diversified residential demand in London using smart meter and demographic data. In 2016 IEEE Power and Energy Society General Meeting (PESGM) (IEEE), pp. 1–5. 10.1109/PESGM.2016.7741076.

[S11] Love, J., Smith, A.Z.P., Watson, S., Oikonomou, E., Summerfield, A., Gleeson, C., Biddulph, P., Chiu, L.F., Wingfield, J., Martin, C., et al. (2017). The addition of heat pump electricity load profiles to GB electricity demand: Evidence from a heat pump field trial. Appl. Energy *204*, 332–342. 10.1016/j.apenergy.2017.07.026.

[S12] Kempton, W., and Tomić, J. (2005). Vehicle-to-grid power fundamentals: Calculating capacity and net revenue. J. Power Sources *144*, 268–279. 10.1016/j.jpowsour.2004.12.025.

[S13] San Román, T.G., Momber, I., Abbad, M.R., and Sánchez Miralles, Á. (2011). Regulatory framework and business models for charging plug-in electric vehicles: Infrastructure, agents, and commercial relationships. Energy Policy *39*, 6360–6375. 10.1016/j.enpol.2011.07.037.

[S14] Muratori, M. (2018). Impact of uncoordinated plug-in electric vehicle charging on residential power demand. Nat. Energy *3*, 193–201. 10.1038/s41560-017-0074-z.

[S15] Kern, T., Dossow, P., and Morlock, E. (2022). Revenue opportunities by integrating combined vehicle-to-home and vehicle-to-grid applications in smart homes. Appl. Energy *307*, 118187. 10.1016/j.apenergy.2021.118187.

[S16] Wulff, N., Steck, F., Gils, H.C., Hoyer-Klick, C., van den Adel, B., and Anderson, J.E. (2020). Comparing Power-System and User-Oriented Battery Electric Vehicle Charging Representation and Its Implications on Energy System

Modeling. Energies *13*, 1093. 10.3390/en13051093.

[S17] Gschwendtner, C., Knoeri, C., and Stephan, A. (2023). The impact of plug-in behavior on the spatial–temporal flexibility of electric vehicle charging load. Sustain. Cities Soc. *88*, 104263. 10.1016/j.scs.2022.104263.

[S18] Schücking, M., Jochem, P., Fichtner, W., Wollersheim, O., and Stella, K. (2017). Charging strategies for economic operations of electric vehicles in commercial applications. Transp. Res. Part Transp. Environ. *51*, 173–189. 10.1016/j.trd.2016.11.032.

[S19] Herb, F. (2010). Alterungsmechanismen in Lithium-Ionen-Batterien und PEM-Brennstoffzellen und deren Einfluss auf die Eigenschaften von daraus bestehenden Hybrid-Systemen. 10.18725/OPARU-1869.

[S20] Pagani, M., Korosec, W., Chokani, N., and Abhari, R.S. (2019). User behaviour and electric vehicle charging infrastructure: An agent-based model assessment. Appl. Energy *254*, 113680. 10.1016/j.apenergy.2019.113680.
